# Pregnancy-Related Deaths in the US, 2018-2022

**DOI:** 10.1001/jamanetworkopen.2025.4325

**Published:** 2025-04-09

**Authors:** Yingxi Chen, Meredith S. Shiels, Tarsicio Uribe-Leitz, Rose L. Molina, Wayne R. Lawrence, Neal D. Freedman, Christian C. Abnet

**Affiliations:** 1 Division of Cancer Epidemiology and Genetics, National Cancer Institute, National Institutes of Health, Rockville, Maryland; 2 Program in Global Surgery and Social Change, Harvard Medical School, Boston, Massachusetts; 3 Department of Plastic Surgery, Boston Children’s Hospital, Boston, Massachusetts; 4 Department of Sport and Health Sciences, Technical University Munich, Epidemiology, Munich, Germany; 5 Harvard Medical School, Boston, Massachusetts; 6 Department Obstetrics and Gynecology, Beth Israel Deaconess Medical Center, Boston, Massachusetts

## Abstract

**Importance:**

The US has the highest rate of preganncy-related death among high-income countries, and disparities continue to widen despite many of these deaths being largely preventable.

**Objective:**

To examine the age-standardized national rates of pregnancy-related death from 2018 to 2022 in the US, stratified by cause, and to compare the rates across state and race and ethnicity.

**Design, Setting, and Participants:**

This serial cross-sectional study used nationwide data on births and pregnancy-related deaths from the Centers for Disease Control and Prevention’s Wide-Ranging Online Data for Epidemiologic Research. All pregnancy-related deaths among women aged 15 to 54 years from 2018 to 2022 were included.

**Exposure:**

State, race and ethnicity, and age.

**Main Outcomes and Measures:**

The primary outcomes were all-cause and cause-specific maternal death and late maternal death (ie, deaths occurring >42 days and up to 1 year after pregnancy). The age-standardized annual and aggregated rate of pregnancy-related mortality (ASR) was estimated by age group and race and ethnicity, and state-specific crude pregnancy-related mortality rates per 100 000 live births and 95% CIs were calculated.

**Results:**

During 2018 to 2022, there were 6283 pregnancy-related deaths, including 1891 late maternal deaths. The ASR increased by 27.7% from 25.3 deaths per 100 000 live births (95% CI, 23.7-26.9) in 2018 to 32.6 deaths per 100 000 live births (95% CI, 31.2-34.8) in 2022. The increase was observed across age group and was disproportionately driven by deaths that occurred among women aged 25 to 39 years (by 36.8%, 2018 vs 2022). There was a considerable variation in rates by state, ranging from 18.5 to 59.7 deaths per 100 000 live births. If the national rate was reduced to the lowest state rate, 2679 pregnancy-related deaths could have been prevented in 2018 to 2022. American Indian and Alaska Native women had the highest ASR (106.3 deaths per 100 000 live births), followed by non-Hispanic Black women (76.9 deaths per 100 000 live births). Although cardiovascular disease was the leading cause of the overall pregnancy-related deaths, cancer, mental and behavior disorders, and drug-induced and alcohol-induced death were important contributing causes of late maternal death.

**Conclusions and Relevance:**

In this cross-sectional analysis of pregnancy-related deaths in the US, rates increased during 2018 to 2022, with large variations by state and race and ethnicity. The concerning rates in the US should be an urgent public health priority.

## Key Points

**Question:**

What is the pattern of pregnancy-related deaths in the US?

**Findings:**

In this cross-sectional analysis of 6283 pregnancy-related deaths in the US from 2018 to 2022, there were large disparities by state and race and ethnicity; American Indian and Alaska Native women had the highest age-standardized annual and aggregated rate (106.3 deaths per 100 000 live births), followed by non-Hispanic Black women (76.9 deaths per 100 000 live births). During 2018 to 2022, 2679 pregnancy-related deaths could have been prevented if the national rate was reduced to the lowest state rate.

**Meaning:**

These findings suggest that detailed, nationwide, age-standardized analyses of pregnancy-related deaths are essential for identifying opportunities to reduce avoidable deaths.

## Teaser

This cross-sectional study examines the age-standardized national rate of pregnancy-related deaths from 2018 to 2022 in the US, by state, race, ethnicity, and age.

## Introduction

In the US, reports from state-based Maternal Mortality Review Committees show that more than 80% of pregnancy-related deaths are preventable.^1^ The Global Burden of Disease study estimated a maternal mortality rate of 26.4 deaths per 100 000 live births in the US in 2015, which was particularly high compared with countries with similar sociodemographic index.^2^ Meanwhile, a recent report from the World Health Organization documented temporal increases in maternal mortality rates in the US.^3^ Yet, data from the National Center for Health Statistics (NCHS) suggest that these documented adverse trends may reflect artifacts of changes in coding practices in recent decades.^4,5^

Measuring the rate of pregnancy-related death is challenging. Currently, 3 government data sources report maternal deaths in the US: the National Vital Statistics System, the Pregnancy-Related Mortality Surveillance System, and state Maternal Mortality Review Committees. These sources often produce differing estimates of rates and trends, with National Vital Statistics System reporting rates more than 50% higher than Pregnancy-Related Mortality Surveillance System.^6^ Despite these discrepancies, the National Vital Statistics System has advantages in timeliness and transparency, whereas Pregnancy-Related Mortality Surveillance System and Maternal Mortality Review Committees typically experience reporting delays.^6^ Studies performed before 2003 used data solely based on death certificate information, resulting in substantial underreporting.^7^ To improve data quality, the NCHS introduced a standard pregnancy checkbox on the revised 2003 death certificate,^5^ although it was not until 2018 when all 50 states and the District of Columbia had fully implemented the revised death certificate and adopted the new coding method.^4^ Since then, the NCHS has resumed routine publication of maternal mortality statistics.^4^ The NCHS reported a maternal death rate of 22.3 per 100 000 live births in 2022, compared with a rate of 32.9 deaths per 100 000 live births in 2021.^8,9^ The results, however, were not age standardized and did not assess late maternal death, defined as deaths that occurred more than 42 days to 1 year after the end of pregnancy.

Disparities in pregnancy-related mortality have been documented in the US. According to data from the Centers for Disease Control and Prevention (CDC), pregnancy-related mortality rate was 2 to 3 times higher in the non-Hispanic Black and American Indian or Alaska Native populations than in the White population.^10^ Similarly, pregnancy-related mortality was 1.7 times higher in rural counties compared with large metropolitan counties.^11^ Nevertheless, most of the estimates relied on data prior to 2018, which may have resulted in underreporting.

In parallel with the United Nation’s Agenda for the Sustainable Development Goals to end preventable maternal deaths by 2030, the American College of Obstetricians and Gynecologists has set policy priorities to eliminate preventable maternal morbidity and mortality.^12,13^ Given the increasing median age at childbirth and the decreasing number of live births observed annually, a more detailed characterization of the current US pregnancy-related death rates with age-standardized estimates would help guide tailored interventions necessary to reduce avoidable pregnancy-related deaths. Therefore, the aim of this study is to describe the patterns of all-cause and cause-specific pregnancy-related mortality rates in the US by state, age group, and race and ethnicity, using data from 2018 to 2022.

## Methods

### Population Cohort and Data Source

In this cross-sectional analysis, we extracted nationwide data of women who delivered a live birth in the US from the Natality database from the CDC Wide-Ranging Online Data for Epidemiologic Research (WONDER). From 2018 to 2022, we identified 18 475 989 live births among women aged 15 to 54 years in the US. We extracted birth data specific to state, age group, race, and ethnicity. We also extracted the age distribution of the mothers in the birth data from 2017 for age standardization. For outcome data, we used the complete US death certificate data from the Multiple Cause of Death database from the CDC WONDER. During the study period, CDC WONDER used the *International Statistical Classification of Diseases and Related Health Problems, Tenth Revision (ICD-10)* coding system. We identified pregnancy-related deaths among women aged 15 to 54 years in the US during the same period. We extracted detailed data on pregnancy-related death among women aged 15 to 54 years reported by year, state, 5-year age group, and race and ethnicity. Death rates were estimated per 100 000 live births. A note on identification of race and ethnicity: race and ethnicity of women were ascertained by the data source.

Institutional review board approval and informed consent were not required as all data used in this study were deidentified and publicly available, in accordance with 45 CFR §46. The study followed the Strengthening the Reporting of Observational Studies in Epidemiology (STROBE) reporting guidelines.^14^

### Outcomes and Variables

The primary outcome of this study was all-cause pregnancy-related death, defined as the death during pregnancy or within 1 year of the end of pregnancy from any cause related to or aggravated by the pregnancy (*ICD-10* codes A34 and O00-O99), on the basis of the updated CDC definition.^15^ This definition included both maternal death, defined as the death of women from any cause related to or aggravated by pregnancy occurring during pregnancy or up to 42 days after the end of a pregnancy, and late maternal death, defined as the death of women from any cause related to or aggravated by pregnancy occurring more than 42 days but less than 1 year after the end of a pregnancy. Secondary outcomes included late maternal death (*ICD-10* code O96) and cause-specific maternal death, defined as hypertensive disorder (*ICD-10* codes O10-O16), abortion (*ICD-10* codes O00-O07), disorder related to pregnancy (*ICD-10* codes O20-O29), complication related to delivery or labor (*ICD-10* codes O30-O48 and O60-O75), and complication related to puerperium (*ICD-10* codes O85-O92). Each specific cause is mutually exclusive to the overall pregnancy-related death. However, this does not include all deaths during pregnancy and exempts incidental causes, including vehicular accidents, suicides, and homicides. Although these deaths could be associated with the fact of the pregnancy, they are not medically related to the pregnancy.

To further understand causes associated with late maternal death, we examined specific disease categories contributing to late maternal death. Major disease categories examined included malignant neoplasm (*ICD-10* codes C00-C97), endocrine disorder (*ICD-10* codes E00-E88), mental and behavioral disorder (*ICD-10* codes F00-F99), cardiovascular disorder (*ICD-10* codes I00-I99), and drug-induced and alcohol-induced death (*ICD-10* codes X40-X44, X60-X64, X85, YU10-Y14, X45, X65, and Y15). Specifically, we extracted mortality data with late maternal death coded as the underlying cause of death and each of the disease category coded as multiple cause of death. Using this approach, the contributory causes were not exclusive, meaning that each late maternal death could have multiple contributory causes.

### Statistical Analysis

We estimated the age-standardized annual and aggregated rate (ASR) of pregnancy-related deaths per 100 000 live births and 95% CIs from 2018 to 2022 using the direct standardization method.^16^ We used US birth data of 5-year age groups in 2017 for standardization. We further estimated crude mortality rates by state. We then used the rates to project the number of pregnancy-related deaths if the nation had the rate of the lowest state during the study period. Specifically, we multiplied the national total number of live births during the study period by the lowest crude mortality rate to estimate the hypothetical number of pregnancy-related deaths. Owing to data confidentiality constraints, state-level pregnancy-related death data of Vermont were not available and, therefore, were not included in state-specific analysis, but data from Vermont were included in the nationwide estimates. In addition, state-specific pregnancy-related death data were not available at the level of detail required for age standardization.

We also estimated aggregated and annual all-cause and cause-specific pregnancy-related mortality rates with 95% CIs for 2018 to 2022, stratified by age group (15-25 years, 25-39 years, and 40-54 years) and for the following race and ethnicity groups: Hispanic or Latino, non-Hispanic American Indian or Alaska Native, non-Hispanic Asian, non-Hispanic Black, and non-Hispanic White. Estimates for other groups could not be included owing to data availability. Age groups and race and ethnicity were first aggregated, respectively, and then age-standardized using the direct standardization method.

Finally, we estimated aggregated late maternal mortality rates associated with the 5 major disease categories (ie, malignant neoplasm, endocrine disorder, mental and behavioral disorder, cardiovascular disorder, and drug-induced and alcohol-induced death) for 2018 to 2022. The analysis was conducted using R statistical software version 4.3.1 (R Project for Statistical Computing).

## Results

### All-Cause Pregnancy-Related Mortality and State-Specific Mortality

In the US, a total of 6283 pregnancy-related deaths, including 1891 late maternal deaths, were reported during 2018 to 2022, yielding an ASR of 33.0 deaths per 100 000 live births (95% CI, 31.2-34.8). The ASR increased by 27.7% from 25.3 deaths per 100 000 live births (95% CI, 23.7-26.9) in 2018 to 32.6 deaths per 100 000 live births (95% CI, 31.2-34.8) in 2022. The increase was partially driven by the increasing late maternal death from an ASR of 7.3 deaths per 100 000 live births (95% CI, 6.4-8.1) in 2018 to 11.1 deaths per 100 000 live births (95% CI, 10.1-12.2) in 2022 (Table). During 2018 to 2022, rates by state varied more than 3-fold, with some southeastern states having higher rates. Specifically, Alabama had the highest crude rate at 59.7 deaths per 100 000 live births (95% CI, 50.8-68.5), followed by Mississippi at 58.2 deaths per 100 000 live births (95% CI, 47.0-69.4). By contrast, California had the lowest rate at 18.5 deaths per 100 000 live births (95% CI, 16.7-20.3), followed by Minnesota at 19.1 deaths per 100 000 live births (95% CI, 14.3-23.8) (Figure; eTable 1 in Supplement 1). If the national rate was the same as the lowest state, 2679 pregnancy-related deaths would have been avoided during 2018 to 2022.

**Table.** Annual Age-Standardized Pregnancy-Related Mortality Rate Per 100 000 Live Births in the US, 2018-2022

Year	Pregnancy-related deaths per 100 000 live births (95% CI)	Late maternal deaths per 100 000 live births (95% CI)^a^
2018	25.3 (23.7-26.9)	7.3 (6.4-8.1)
2019	28.7 (27.0-30.3)	8.4 (7.5-9.3)
2020	34.6 (32.7-36.4)	11.0 (10.0-12.1)
2021	44.1 (42.0-46.2)	12.1 (11.0-13.2)
2022	32.6 (30.8-34.8)	11.1 (10.1-12.2)

^a^ Late maternal deaths are defined as those occurring more than 42 days and up to 1 year after pregnancy.

**Figure.**
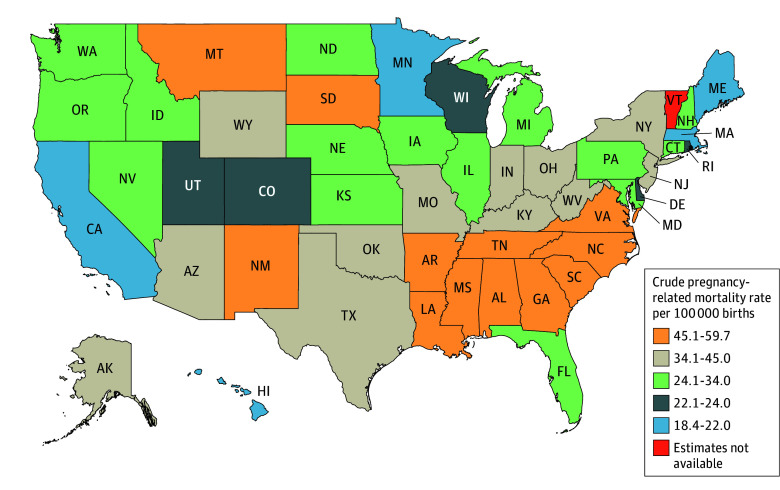
Aggregated Crude Pregnancy-Related Mortality Rate Per 100 000 Live Births, by States, 2018-2022

### All-Cause Pregnancy-Related Mortality by Age Group, Race, and Ethnicity

During the study period, women aged 25 to 39 years had the highest increase in rate by 36.8% (ASR in 2018 vs 2022, 24.5 vs 33.5 deaths per 100 000 live births; 4500 women), followed by those aged 15 to 24 years by 25.8% (ASR in 2018 vs 2022, 21.0 vs 16.7 deaths per 100 000 live births; 870 deaths). Although the absolute rates in this age group were much higher, women aged 40 to 54 years had a smaller 7.4% increase in mortality rate (ASR in 2018 vs 2022, 121.0 vs 112.6 deaths per 100 000 live births; 913 deaths).

There was a nearly 5-fold difference in pregnancy-related mortality rates by racial and ethnic groups. Compared with other racial and ethnic groups, American Indian and Alaska Native women had markedly higher mortality rate (ASR, 106.3 deaths per 100 000 live births; 95% CI, 103.1-109.6; 125 deaths), which was 3.8 times higher than the rate among White women. Non-Hispanic Black women had the second highest rate (ASR, 76.9 deaths per 100 000 live births; 95% CI, 74.2-79.7; 1986 deaths), which was 2.8 times higher than the rate among White women (ASR, 27.6 deaths per 100 000 live births; 95% CI, 26.0-29.3; 2691 deaths). Latino or Hispanic women (ASR, 25.9 deaths per 100 000 live births; 95% CI, 24.3-27.5; 1137 deaths) and non-Hispanic Asian women (ASR, 21.8 deaths per 100 000 live births; 95% CI, 20.3-23.2; 224 deaths) had the lowest rates.

### Cause-Specific Maternal Mortality by Race and Ethnicity

By underlying causes, other disorders predominantly related to pregnancy (a category that includes hemorrhage, venous complications, and so forth) had the highest rate and accounted for 17.4% of the overall pregnancy-related deaths (ASR, 5.9 deaths per 100 000 live births; 95% CI, 5.6-6.3; 1094 deaths), followed by delivery and labor (ASR, 2.2 deaths per 100 000 live births; 95% CI, 2.0-2.4; 403 deaths), complications related to puerperium (ASR, 2.0 deaths per 100 000 live births; 95% CI, 1.8-2.2; 369 deaths), and hypertensive disorder (ASR, 2.0 deaths per 100 000 live births; 95% CI, 1.8-2.2; 365 deaths). Together, hypertensive disorder and disorders related to pregnancy accounted for more than 20% of deaths. Compared with other racial and ethnic groups, non-Hispanic Black women had the highest mortality rate for each specific cause examined, except for deaths due to disorders related to pregnancy, for which American Indian and Alaska Native women had the highest rate (ASR, 20.6 deaths per 100 000 live births; 95% CI, 13.0-28.2; 28 deaths), and deaths related to delivery or labor, for which non-Hispanic Asian women had the highest rate (ASR, 3.4 deaths per 100 000 live births; 95% CI, 2.4-4.5; 39 deaths) (eTable 2 in Supplement 1).

### Late Maternal Death and Contributing Causes

Late maternal deaths accounted for 30% of the overall pregnancy-related mortality (ASR, 10.0 deaths per 100 000 live births; 95% CI, 9.0-11.0; 1891 deaths). American Indian and Alaska Native women had the highest rate (ASR, 32.3 deaths per 100 000 live births; 95% CI, 22.8-41.9; 44 deaths), followed by non-Hispanic Black women (ASR, 22.0 deaths per 100 000 live births; 95% CI, 20.2-23.8; 584 deaths). Late maternal death, disorders related to pregnancy, and hypertensive disorders accounted for more than 60% of the overall deaths in these groups. The rates for other racial and ethnic groups were lower, with ASRs of 8.7 deaths per 100 000 live births (95% CI, 8.1-9.3; 818 women) among non-Hispanic White women, 7.8 deaths per 100 000 live births (95% CI, 7.0-8.6; 348 women) among Latino or Hispanic women, and 5.8 deaths per 100 000 live births (95% CI, 4.4-7.2; 66 women) among non-Hispanic Asian women.

To understand major causes associated with late maternal death, we further analyzed contributing conditions. Cardiovascular disorder was the leading contributor (ASR, 4.7 deaths per 100 000 live births; 95% CI, 4.4-5.0; 869 deaths), followed by malignant neoplasm (ASR, 2.0 deaths per 100 000 live births; 95% CI, 1.8-2.2; 361 deaths); cancer accounted for almost 20% of late maternal deaths. Other important contributors included endocrine disorder (ASR, 1.6 deaths per 100 000 live births; 95% CI, 1.4-1.8; 295 deaths), drug-induced and alcohol-induced death (ASR, 1.1 deaths per 100 000 live births; 95% CI, 1.0-1.3; 211 deaths), and mental and behavior disorder (ASR, 1.0 deaths per 100 000 live births; 95% CI, 0.9-1.2; 190 deaths). Mental and behavior disorders and drug-and alcohol-induced death contributed to 21.2% of late maternal deaths.

## Discussion

In this cross-sectional study, rates of pregnancy-related death increased by 27.7% in the US between 2018 and 2022, but there were nonmonotonic patterns, and the highest rate was in the year 2021. The increase was observed across age group with a disproportionate increase of 36.8% among women aged 25 to 39 years. If the nation had achieved the lowest state rate estimated here, 2679 pregnancy-related deaths could have been prevented during the study period. Additionally, the pregnancy-related death rate was 3.8 times higher among American Indian and Alaska Native women and 2.8 times higher among non-Hispanic Black women compared with the rate among non-Hispanic White women. Although cardiovascular disease was the leading cause of the overall pregnancy-related death, cancer, drug-induced and alcohol-induced death, and mental and behavior disorders are important contributing causes of late maternal death.

Our estimates of maternal death rates included both maternal death and late maternal death using the standard definition of death during pregnancy or within 1 year of the end of pregnancy from any cause related to or aggravated by the pregnancy. The trend aligns with recent US estimates, where rates increased from 2018, peaked in 2021, and then decreased in 2022.^8,9^ Nevertheless, the rate in 2022 remained markedly higher compared with that in 2018. Women aged 25 to 39 years accounted for most pregnancy-related deaths and experienced the highest increase. Late maternal death has not been considered in the World Health Organization definition of pregnancy-related mortality.^17^ However, late maternal death occurs in what could be a health care delivery gap between obstetric care and transition to primary care. Measuring the rate of late maternal death is essential for a comprehensive understanding of the long-term health outcomes of pregnancy to identify opportunities to prevent avoidable pregnancy-related deaths and reduce disparities.

During 2018 to 2022, we observed persistent disparities in pregnancy-related death among American Indian and Alaska Native women and non-Hispanic Black women. Late maternal death, disorders related to pregnancy, and hypertensive disorders accounted for more than 60% of the overall deaths in these groups. Previous US data suggested persistent disparities among American Indian and Alaska Native and Black persons vs Asian, Native Hawaiian, Other Pacific Islander, Hispanic, and White persons.^18,19^ These disparities do not appear to have improved over time.^18^ In the current analysis, we found particularly higher mortality rates due to late maternal death among American Indian and Alaska Native women and non-Hispanic Black women, indicating that these groups may face disparities in access to postnatal care, as well as other socioeconomic and systemic challenges impacting maternal health outcomes.

Moreover, our cause-specific analyses indicated that mental and behavior disorders and drug and alcohol induced death contributed to 21.2% of late maternal deaths. A similar analysis from the United Kingdom and Ireland suggested that psychiatric causes led to almost a quarter of late maternal deaths during 2009 to 2014.^20^ Maternal anxiety and depression are the most common complications of childbirth.^21^ According to the American Psychological Association, the prevalence of depression ranges from 8.5% to 11.0% during pregnancy and from 6.5% to 12.9% during the first year post partum.^22^ It is critical to address mental health needs as part of efforts to reduce pregnancy-related death.

In the US, homicide, suicide, and drug overdose are the leading causes of pregnancy-associated death, which include pregnancy-related death and death from incidental causes while pregnant.^23,24,25^ Although the current analysis does not analyze maternal death from incidental causes, published data show that intimate partner violence during pregnancy is an important cause of maternal death.^26^ Moreover, recent US statistics indicate a noteworthy increase in pregnancy-associated homicides in 2020.^27^ Traditionally, pregnancy-associated deaths from incidental causes have not been considered in maternal deaths, so we did not include them in our estimates. Yet, these deaths are important public health concerns.^28^

The current analysis confirmed cardiovascular disease as an important cause of pregnancy-related death. Pregnancy can affect the cardiovascular system, leading to cardiovascular disease (eg, hypertensive disorder), aggravating underlying conditions (eg, pulmonary arterial hypertension), or cause-specific disease (eg, peripartum cardiomyopathy).^29^ Some of these causes, such as hypertensive disorder, are considered a direct cause of maternal death, while others are categorized as disorders related to pregnancy (eg, venous complications or thrombosis). In the current analysis, more than 20% of maternal deaths were directly attributable to hypertensive disorder and disorders related to pregnancy, emphasizing the importance of cardio-obstetric care during and after pregnancy.

Although cancer has not been considered a direct cause of pregnancy-related death, cancer was the second leading contributor of late maternal death in the US, associated with almost 20% of the deaths. Cancer is the leading cause of death in the general population, although pregnancies complicated by cancer are potential threats to maternal well-being. Published US data suggested that more than 70% of pregnancy-associated cancers were diagnosed during the first year post partum.^30^ Increasing maternal age leads to higher cancer risks and highlights the importance of greater awareness regarding the occurrence and recurrence of cancer during pregnancy and the postpartum period. Maternal deaths due to cancer can be due to excess incidence during pregnancy (eg, breast cancer risk is elevated during and shortly after pregnancy^31^) or could be due to voluntary or involuntary changes in treatment while pregnant. Our study period straddled the several years of the COVID-19 pandemic. Although COVID-19 itself would not be coded as a pregnancy-related cause of death, its impact on maternal health and disruptions to the health care system likely contributed to the increased pregnancy-related mortality rates, particularly in 2021.

### Strengths and Limitations

A strength of this study was the age standardization and stratification of analysis by state, age group, and racial and ethnic group, enabling a comprehensive understanding of the growing scope of pregnancy-related death in the US. Another strength of this study was the detailed analysis of cause-specific death by direct and indirect obstetric cause and cause-associated late maternal death, providing important information for potential opportunities to reduce preventable pregnancy-related death and disparities.

However, this study also has limitations that should be mentioned. There have been concerns about the quality of pregnancy-related death data, including the effects of introducing the pregnancy box to the death certificate data, changing in coding practice, potential undercounting of early postpartum deaths and overcounting of late maternal deaths due to death certificate questions on timing, and potential misclassification for pregnancy status. To address this limitation, we restricted study population to women aged 15 to 54 years and the study period to 2018 and after, when the implementation of the revised certificate was completed and a new coding method was adopted to mitigate probable coding errors, but coding errors may still be present.^4^ Another important limitation was use of death certificate data, which may misclassify race and ethnicity because such data on death certificates are not self-reported. Furthermore, owing to the nature of the data, we were only able to assess diseases associated with late maternal deaths, and the actual causes of late maternal deaths at the national level were difficult to measure because of data availability. Regardless, considering the importance of preventing avoidable pregnancy-related death, assessing causes associated with late maternal death can still provide valuable perspective. Moreover, because of small numbers in some subgroups and data availability, age-standardized estimates of states were not available, and rates of other racial and ethnic groups could not be included, limiting the interpretation of the study results. Furthermore, owing to the descriptive nature of the study, we are unable to analyze individual-level factors contributing to the observed disparities.

## Conclusions

Pregnancy-related death is a major public health concern in the US. In this cross-sectional analysis, we estimate that 2679 pregnancy-related deaths during 2018 to 2022 could have been prevented if the national rate were reduced to the lowest state rate. American Indian and Alaska Native women and non-Hispanic Black women continue to experience particularly high rates compared with other racial and ethnic groups. Our analysis provides important insights that should inform the shared goal of preventing avoidable pregnancy-related deaths in the US.

## Notes

Supplement 1. **eTable 1.** Aggregated pregnancy-related mortality rate per 100,000 live births, by state, 2018-2022 **eTable 2.** Aggregated cause-specific pregnancy-related mortality rate per 100,000 live births, by age group and race and ethnicity, 2018-2022

Supplement 2.
Data Sharing Statement
