# Supplementary material for: Pregnancy-Related Deaths in the US, 2018-2022
Source: JAMA Netw Open. 2025 Apr 9;8(4):e254325. doi: 10.1001/jamanetworkopen.2025.4325 (PMC11983229; doi:10.1001/jamanetworkopen.2025.4325)
Supplement: Supplement 1. — eTable 1. Aggregated pregnancy-related mortality rate per 100,000 live births, by state, 2018-2022 eTable 2. Aggregated cause-specific pregnancy-related mortality rate per 100,000 live births, by age group and race and ethnicity, 2018-2022 [file jamanetwopen-e254325-s001.pdf]

Supplemental Online Content

Chen Y, Shiels MS, Urbe-Leitz T, et al. Pregnancy-related deaths in the US, 2018-2022. *JAMA Netw Open*. 2025;8(4):e254325. doi:10.1001/jamanetworkopen.2025.4325

eTable 1. Aggregated pregnancy-related mortality rate per 100,000 live births, by State, 2018-2022

eTable 2. Aggregated cause-specific pregnancy-related mortality rate per 100,000 live births, by age group and race and ethnicity, 2018-2022

This supplemental material has been provided by the authors to give readers additional information about their work.

© 2025 Chen Y et al. *JAMA Network Open*.

eTable 1. Aggregated pregnancy-related mortality rate per 100,000 live births, by State, 2018-2022

State	Deaths (N)	Rate per 100,000	Standard error	Lower condifence interval	Higher confidence interval
California	399	18.5	0.9	16.7	20.3
Minnesota	62	19.1	2.4	14.3	23.8
Hawaii	16	19.8	5.0	10.1	29.5
Maine	12	20.1	5.8	8.7	31.5
Massachusetts	73	21.3	2.5	16.4	26.2
Delaware	12	22.7	6.6	9.9	35.6
Colorado	71	22.7	2.7	17.4	28.0
Utah	53	22.8	3.1	16.7	29.0
Wisconsin	71	22.9	2.7	17.6	28.3
Rhode Island	12	23.3	6.7	10.1	36.5
Illinois	165	24.3	1.9	20.6	28.0
Nevada	42	24.5	3.8	17.1	31.9
New Hampshire	15	24.9	6.4	12.3	37.5
Washington	109	25.9	2.5	21.0	30.7
North Dakota	14	27.6	7.4	13.1	42.0
Iowa	51	27.6	3.9	20.0	35.2
Pennsylvania	184	27.7	2.0	23.7	31.8
Connecticut	50	28.8	4.1	20.8	36.8
Oregon	61	29.9	3.8	22.4	37.4
Nebraska	37	30.0	4.9	20.3	39.6
Idaho	34	31.0	5.3	20.6	41.4
Maryland	108	31.2	3.0	25.3	37.0
Florida	348	31.9	1.7	28.5	35.2
Michigan	174	32.9	2.5	28.0	37.8
Kansas	59	33.7	4.4	25.1	42.3
Oklahoma	84	34.5	3.8	27.2	41.9
New York	375	34.9	1.8	31.3	38.4

© 2025 Chen Y et al. *JAMA Network Open*.

Alaska	17	35.4	8.6	18.5	52.2
Ohio	242	36.9	2.4	32.2	41.5
Texas	700	37.1	1.4	34.4	39.9
New Jersey	190	37.8	2.7	32.4	43.1
Wyoming	12	38.1	11.0	16.5	59.6
Indiana	153	38.2	3.1	32.1	44.3
Missouri	137	38.8	3.3	32.3	45.3
Arizona	159	40.4	3.2	34.1	46.7
West Virginia	38	43.3	7.0	29.5	57.1
Kentucky	116	44.1	4.1	36.1	52.1
New Mexico	50	45.1	6.4	32.6	57.6
Virginia	221	45.7	3.1	39.7	51.8
South Carolina	134	47.1	4.1	39.2	55.1
North Carolina	285	47.8	2.8	42.3	53.4
District of Columbia	21	47.9	10.4	27.4	68.3
Montana	27	48.4	9.3	30.2	66.7
Louisiana	146	50.4	4.2	42.3	58.6
Arkansas	93	51.6	5.4	41.1	62.1
Georgia	325	52.0	2.9	46.4	57.7
South Dakota	30	52.8	9.6	33.9	71.7
Tennessee	218	54.0	3.7	46.8	61.2
Mississippi	104	58.2	5.7	47.0	69.4
Alabama	173	59.7	4.5	50.8	68.5
Vermont*	ND	ND	ND	ND	ND

ND: Not determined.

*Deaths values are suppressed due to small numbers, but the deaths values are included in the nation-wide analysis.

© 2025 Chen Y et al. *JAMA Network Open*.

eTable 2. Aggregated cause-specific pregnancy-related mortality rate per 100,000 live births, by age group and race and ethnicity, 2018-2022

Specific causes	Deaths (N)	Rate per 100,000	Standard error	Lower condifence interval	Higher confidence interval	Proportion of overall pregnancy-related deaths (%)
**Total disorder related to pregancy**	**1094**	**5.9**	**0.2**	**5.6**	**6.3**	17%
**Age group**						
15-24 year-old	148	3.5	0.3	3.0	4.1	-
25-39 year-old	753	5.5	0.2	5.1	5.9	-
40-54 year-old	193	28.9	2.1	24.9	33.0	-
**Race and ethnicity**						
American Indian and Alaska Native	28	20.6	3.9	13.0	28.2	
Non-Hispanic Asian	28	2.5	0.5	1.6	3.4	
Non-Hispanic Black	337	12.7	0.7	11.3	14.0	-
Non-Hispanic White	486	5.1	0.2	4.7	5.6	-
Latino/Hispanic	193	4.3	0.3	3.7	4.9	-
**Total hypertensive disorders**	**365**	**2.0**	**0.1**	**1.8**	**2.2**	6%
**Age group**						
15-24 year-old	44	1.1	0.2	0.7	1.4	-
25-39 year-old	253	1.9	0.1	1.6	2.1	-
40-54 year-old	68	10.2	1.2	7.8	12.6	-
**Race and ethnicity**						
American Indian and Alaska Native	NA					
Non-Hispanic Asian	13	1.1	0.3	0.5	1.8	-
Non-Hispanic Black	161	6.1	0.5	5.1	7.0	-
Non-Hispanic White	124	1.3	0.1	1.1	1.5	-
Latino/Hispanic	58	1.3	0.2	1.0	1.6	-
**Total delivery or labor related**	**403**	**2.2**	**0.1**	**2.0**	**2.4**	6%
**Age group**						

© 2025 Chen Y et al. *JAMA Network Open*.

15-24 year-old	42	1.0	0.2	0.7	1.3	-
25-39 year-old	314	2.3	0.1	2.0	2.6	-
40-54 year-old	47	7.0	1.0	5.0	9.1	-
**Race and ethnicity**						
American Indian and Alaska Native	NA					
Non-Hispanic Asian	39	3.4	0.6	2.4	4.5	-
Non-Hispanic Black	88	3.3	0.4	2.6	4.0	-
Non-Hispanic White	164	1.7	0.1	1.5	2.0	-
Latino/Hispanic	92	2.1	0.2	1.6	2.5	-
**Total puerperium**	**369**	**2.0**	**0.1**	**1.8**	**2.2**	6%
**Age group**						
15-24 year-old	51	1.2	0.2	0.9	1.6	-
25-39 year-old	266	2.0	0.1	1.7	2.2	-
40-54 year-old	52	7.8	1.1	5.7	9.9	-
**Race and ethnicity**						
American Indian and Alaska Native	NA					-
Non-Hispanic Asian	23	2.0	0.4	1.2	2.9	-
Non-Hispanic Black	138	5.2	0.4	4.3	6.1	-
Non-Hispanic White	146	1.5	0.1	1.3	1.8	
Latino/Hispanic	51	1.1	0.2	0.8	1.5	-
**Total abortion**	**162**	**0.9**	**0.1**	**0.7**	**1.0**	3%
**Age group**						
15-24 year-old	38	0.9	0.1	0.6	1.2	-
25-39 year-old	107	0.8	0.1	0.6	0.9	-
40-54 year-old	17	2.5	0.6	1.3	3.8	-
**Race and ethnicity**						
American Indian and Alaska Native	NA					
Non-Hispanic Asian	NA					-
Non-Hispanic Black	63	2.4	0.3	1.8	3.0	-
Non-Hispanic White	48	0.5	0.1	0.4	0.7	-
Latino/Hispanic	38	0.9	0.1	0.6	1.1	-

© 2025 Chen Y et al. *JAMA Network Open*.
