# Supplementary material for: Pregnancy-Related Deaths in the US, 2018-2022
Source: JAMA Netw Open. 2025 Apr 9;8(4):e254325. doi: 10.1001/jamanetworkopen.2025.4325 (PMC11983229; doi:10.1001/jamanetworkopen.2025.4325)
Supplement: Supplement 2. — Data Sharing Statement [file jamanetwopen-e254325-s002.pdf]

# Data Sharing Statement

Chen. Pregnancy-Related Deaths in the US, 2018-2022. *JAMA Netw Open*. Published April 09, 2025. doi:10.1001/jamanetworkopen.2025.4325

**Data**
**Data available:** Yes
**Data types:** Deidentified participant data
**How to access data:** Data are publicly available at :https://wonder.cdc.gov/
**When available:** With publication

**Supporting Documents**
**Document types:** None

**Additional Information**
**Who can access the data:** Researchers whose proposed use of the data has been approved
**Types of analyses:** For research purpose.
**Mechanisms of data availability:** Without investigator support
